# Cholangiolytic Changes in Statin-Induced Liver Injury

**DOI:** 10.1155/2020/9650619

**Published:** 2020-02-13

**Authors:** Preethi Dileep Menon, Tamneet Singh, Hopethe Hubbard, Sarah Hackman, Francis E. Sharkey

**Affiliations:** ^1^Department of Pathology and Laboratory Medicine, UT Health San Antonio, 7703 Floyd Curl Drive, San Antonio, Texas 78229-3900, USA; ^2^Division of Gastroenterology and Nutrition, UT Health San Antonio, 7703 Floyd Curl Drive, San Antonio, Texas 78229, USA

## Abstract

Atorvastatin is a commonly used oral cholesterol-lowering agent. Side effects associated with statin therapy include arthralgia, myalgia, dyspepsia, weakness, and headache. Prospective and retrospective studies of drug-induced liver injury have identified statin-induced hepatotoxicity, with atorvastatin being the most commonly cited. Associated liver function test elevations have varied from hepatocellular to cholestatic/mixed pattern. We report a case of a 58-year-old woman that illustrates unusual histologic findings associated with a mixed pattern of statin-induced liver injury. While being treated with atorvastatin, the patient exhibited repeated bouts of abdominal pain over a year associated with biliary tree dilation, variably attributed to postcholecystectomy dilation and stenosis of the ampulla of Vater. Following sphincterotomy, the patient's bilirubin normalized but the other liver function tests remained elevated. Liver biopsy revealed portal and lobular inflammation with cholangiolysis. The patient's liver function tests normalized following cessation of atorvastatin therapy.

## 1. Introduction

Atorvastatin is a commonly used oral cholesterol-lowering agent. Like other statins, it is used for reducing the risk of atherosclerosis and its complications, such as myocardial infarction and stroke [1–4]. Common side effects associated with statin therapy include arthralgia, myalgia, dyspepsia, weakness, and headache. In early clinical trials, elevations of liver enzymes were observed in up to 2% of patients; however, clinically apparent liver injury was rarely observed [1–7]. In the majority of cases, these mild elevations in liver enzymes were not clinically significant and rarely required discontinuation of therapy [8, 9]. Drug-induced liver injury (DILI) can be defined as injury caused by usual doses of drugs, herbal products, and dietary supplements [10]. Major prospective and retrospective studies of drug-induced liver injury have implicated statin-suspected hepatotoxicity [11–17]. Atorvastatin has been among the most commonly associated [11–13, 18], with liver function test elevations varying from hepatocellular to cholestatic/mixed pattern [19]. This case report illustrates unusual histologic findings associated with a mixed pattern of statin-induced liver injury.

## 2. Case Presentation

A 58-year-old Hispanic woman with a BMI of 27.6 and a clinical history of diabetes mellitus type 2, hypertension, and hyperlipidemia, and a prior surgical history of cholecystectomy, presented with acute onset of epigastric pain.

Upon admission, laboratory tests were notable for elevations in alanine aminotransferase (ALT) (748 U/L; reference range < 36 U/L), aspartate aminotransferase (AST) (325 U/L; reference range < 32 U/L), alkaline phosphatase (ALP) (826 U/L; reference range 45-117 U/L), and total bilirubin (1.5 mg/dL; reference range 0.2-1.2 mg/dL), consistent with a mixed hepatocellular/cholestatic pattern of liver injury. A viral hepatitis panel was negative for hepatitis A, hepatitis B, hepatitis C, hepatitis E, and human immunodeficiency virus (HIV). Antinuclear antibody (ANA), antimitochondrial antibody (AMA), and anti-smooth muscle antibody (ASMA) tests were also negative. Elevations were noted in serum immunoglobulins, as follows: IgG (2700 mg/dL; reference range: 650-1600 mg/dL), IgG1 (1600 mg/dL; reference range: 240-1118 mg/dL), and IgG2 (574 mg/dL; reference range: 124-549 mg/dL). However, IgG3 (86 mg/dL; reference range: 21-134 mg/dL) and IgG4 (83 mg/dL; reference range: 1-123 mg/dL) were within normal limits. Computed tomography (CT) scan of the abdomen revealed a normal-appearing liver and mild intrahepatic/extrahepatic biliary dilation up to 1.0 cm, deemed to be a normal compensatory response after cholecystectomy. Since no discernable cause was found for her laboratory test findings, she was discharged.

The patient continued to have recurrent abdominal pain over the course of a year and subsequently developed jaundice, fever, and a pigmented, purpuric, and pruritic dermatosis overlying her face and bilateral lower extremities. Subsequent laboratory tests revealed continued elevations in AST (503 U/L), ALT (1026 U/L), ALP (446 U/L), and total bilirubin (5.0 mg/dL). CT scan of the abdomen again reported a normal-appearing liver with dilation of the intrahepatic ducts and common bile duct up to 1.0 cm, again deemed to be related to postcholecystectomy status. Due to ongoing abdominal pain and jaundice, she underwent endoscopic retrograde cholangiopancreatography (ERCP) which noted dilated intrahepatic and extrahepatic bile ducts and a stenotic papilla. The latter finding was thought to be the source of her abdominal pain and abnormal liver enzymes (Table 1), and she subsequently underwent a sphincterotomy. Four weeks later, her bilirubin had normalized; however, there was no significant relief of her symptoms, and liver enzymes remained elevated (Table 1). An endoscopic ultrasound (EUS) and repeat ERCP reported a common bile duct up to 1 cm, normal intrahepatic bile ducts, no filling defects, and an otherwise normal exam.

A liver biopsy was then performed, which was initially interpreted as showing nonspecific portal inflammation with edema and focal ductular reaction. Upon further review, the possibility of a drug-induced liver injury (DILI) related to statins was raised. Her medication list was reviewed, and she was advised to discontinue atorvastatin, a medication she had been taking for several years prior to her initial presentation. Liver function tests and hepatic ultrasound prior to beginning atorvastatin were not available. Within 4 weeks after discontinuation, her liver enzymes downtrended to AST 24 U/L, ALT 51 U/L, and ALP 179 U/L. Within 12 weeks of discontinuation, her rash resolved, abdominal pain significantly improved, and liver enzymes normalized (ALT 27 U/L, AST 17 U/L, and ALP 110 U/L) (Table 1).

## 3. Pathology

**Figure 1:**
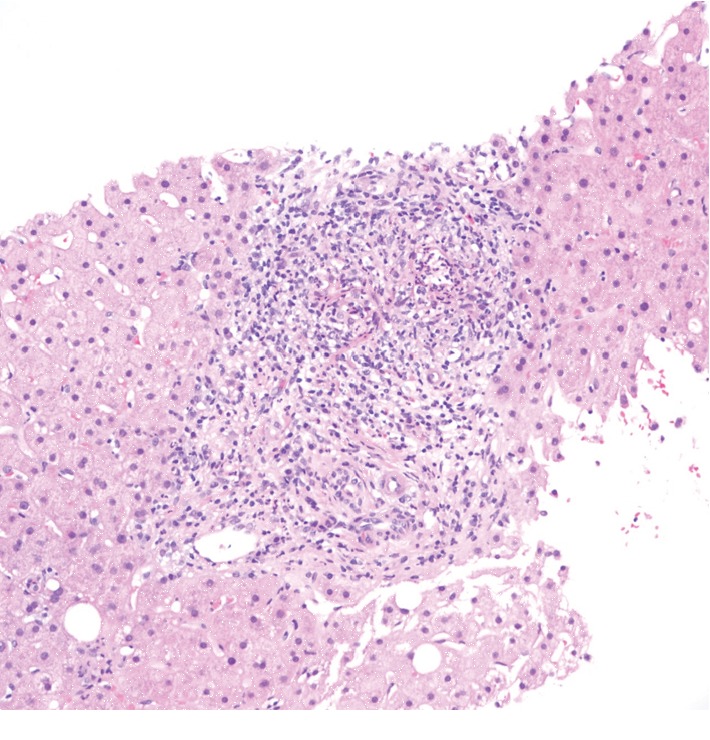
Dense portal inflammation (hematoxylin and eosin, 200x).

**Figure 2:**
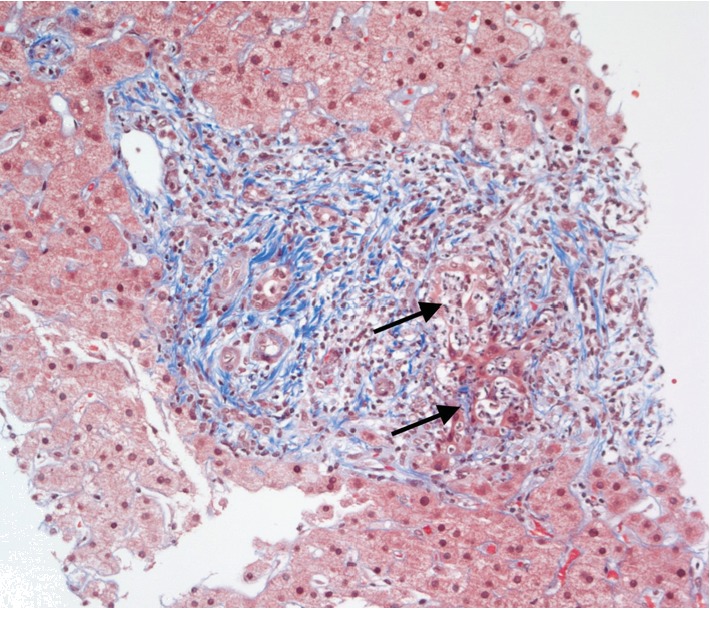
Mild portal fibrosis and neutrophilic cholangiolysis (arrow) (Masson trichrome, 250x).

The portal tracts were generally expanded by a moderate to focally marked mixed inflammatory cell infiltrate that included neutrophils, eosinophils, and plasma cells (Figure 1). Focal interface activity was present, but plasma cells were not involved. Many portal tracts displayed increased numbers of well-formed bile ducts and cholangioles distributed throughout the portal tract (Figure 2). There was a tendency for the portal inflammatory cell infiltrates to show greater density around bile ducts, and there were several foci of bizarrely proliferated bile ducts showing degenerative changes (cholangiolysis), with both intramural and intraluminal neutrophils (Figure 2). Trichrome stain showed minimally increased portal fibrosis without bridging (Figure 2).

**Figure 3:**
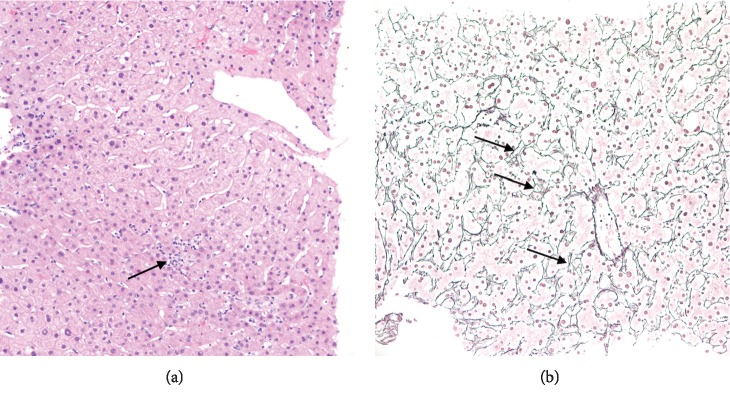
(a) Lobular parenchyma with focal mononuclear cell infiltrate (arrow) (hematoxylin and eosin, 200x). (b) Multifocal reticulum fiber collapse (arrows) (reticulin stain, 200x).

The lobular parenchyma displayed mild disarray, with scattered foci of mononuclear cell infiltrates, but acidophil bodies were not seen, and cholestasis-related changes (e.g., feathery degeneration) were absent (Figure 3(a)). A reticulin stain showed multifocal collapse (Figure 3(b)). There was neither steatosis nor hepatocyte ballooning, and no pericellular fibrosis was seen by trichrome stain.

## 4. Discussion

Most reports of statin-associated liver injury have been associated with atorvastatin [11–13, 18–20]. The Swedish Hepatotoxicity Registry indicates that statin-induced liver injury has caused significant alkaline phosphatase (ALP) levels (3x upper limit of normal) more frequently than other forms of drug-induced liver injury [19]. Björnsson [21] reviewed three prospective drug-induced liver injury studies that were undertaken in Spain, the United States, and Iceland. In the US study, the majority (55%) of cases of statin-related liver injury occurred within 6 months of beginning treatment, with the remainder presenting later. The type of liver injury seemed to vary widely among these studies and included hepatocellular, cholestatic, and mixed liver injury patterns. A mixed pattern was more common with atorvastatin-induced injury (56% of patients) than with simvastatin-induced liver injury (24% of patients) [19].

In our case, the liver biopsy showed a mixed inflammatory cell infiltrate expanding the portal triad and—most strikingly—neutrophilic destruction of bile ducts. The latter finding may be seen in biliary obstruction, ascending cholangitis, sepsis, cholangiolytic drug reactions, and hyperalimentation [22, 23]. Li et al. conducted a detailed histologic analysis of liver biopsies with biliary tract disorders and emphasized that the appropriate diagnosis relies on clinical data in addition to pathomorphology [24]. In the current case, the patient had abdominal pain and liver biopsy findings suggestive of biliary tract obstruction, but the abdominal pain recurred multiple times during the course of a year, and the patient never had either fever or jaundice—i.e., the other two findings that make up the Charcot triad that is diagnostic of acute cholangitis.

Following sphincterotomy, the bilirubin level normalized to the degree expected [23], but the liver enzymes continued elevated [24], with a mixed pattern. This, plus the absence of cholestasis-related changes in the lobular parenchyma and the distribution of bile ducts and cholangioles in the portal tracts (i.e., not predominantly at the edges of the portal tracts), indicated that something more than biliary obstruction was present in this patient, and nonbiliary causes were considered.

Neither metabolic syndrome nor chronic alcoholic liver disease seems likely since neither would raise the transaminases and alkaline phosphatase levels to such high levels as was seen in our patient. Their characteristic histologic features (i.e., steatosis, hepatocyte ballooning, and lobular fibrosis) were lacking, and neither is known to cause cholangiolytic changes in bile ducts. The laboratory findings helped to exclude viral hepatitis, chronic infections, and autoimmune etiologies.

Alla et al. [25] reported 6 cases of autoimmune hepatitis induced by statins. Five of the six cases were attributed to atorvastatin. However, autoimmune markers in the current case, including anti-smooth muscle antibody (ASMA), antinuclear antibody (ANA), antimitochondrial antibody (AMA), and soluble liver antigen antibody IgG (SLA-IgG), were negative, leaving autoimmune cholangitis as unlikely in this patient. The viral hepatitis markers were also negative, and markers for malignancy including CA19.9 and CEA were negative as well. Therefore, after excluding other known causes of cholangiolysis and observing the improvement in serum liver function tests and other clinical features upon discontinuation of atorvastatin, the diagnosis of atorvastatin-induced liver injury appears to be well-supported despite the unusual morphology.

## Figures and Tables

**Table 1:** Trend of liver function tests before and after discontinuation of atorvastatin.

	At time of sphincterotomy	4 weeks post sphincterotomy	Prior to biopsy (2 weeks later)	12 weeks after discontinuing atorvastatin
Total bilirubin (mg/dL)	2.6	0.7	0.7	0.6
ALT (U/L)	544	225	211	27
AST (U/L)	255	237	119	17
ALP (U/L)	554	971	867	110
Albumin (g/dL)	2.9	2.9	3.3	3.5
INR	—	1.0	—	1.0
